# CD4 T-Cell Dysregulation in Psoriatic Arthritis Reveals a Regulatory Role for IL-22

**DOI:** 10.3389/fimmu.2017.01403

**Published:** 2017-10-27

**Authors:** Amara Ezeonyeji, Helen Baldwin, Milica Vukmanovic-Stejic, Michael R. Ehrenstein

**Affiliations:** ^1^Centre for Rheumatology, Division of Medicine, University College London, London, United Kingdom; ^2^Division of Infection and Immunity, University College London, London, United Kingdom

**Keywords:** IL-22, interferon-gamma, naïve T cell, psoriatic arthritis, anti-TNF

## Abstract

Dysregulation of interleukin-22 (IL-22) has been associated with autoimmune diseases but divergent effects upon inflammation have hampered efforts to define its contribution to pathogenesis. Here, we examined the role of IL-22 in patients with psoriatic arthritis (PsA). In the peripheral blood of PsA patients, there was a decrease in IL-22^+^CD4^+^ T cells compared with healthy controls resulting in a heightened CD4^+^ IFNγ^+^/IL-22^+^ ratio accompanied by diminished CCR6 expression. IL-22 expressing cells were depleted primarily from the central memory CD4 T-cell subset in PsA patients. Paradoxically IL-22 and particularly interferon-gamma (IFNγ) production were elevated within a CD4^+^ T-cell subset with phenotypic markers characteristic of naïve T cells (CD3^+^CD4^+^CD27^+^CD45RA^+^CCR7^+^CD95^−^IL-2Rβ^−^) from PsA patients with the highest IFNγ^+^/IL-22^+^ ratio of all the CD4 subsets. These unconventional “naïve” CD4^+^ T cells from PsA patients displayed some phenotypic and functional characteristics of memory cells including a marked proliferative response. Increased IFNγ production from these unconventional “naïve” T cells from PsA patients promoted greater expression of the chemo-attractant CXCL9 by HaCaT keratinocytes compared with their healthy counterparts. Treatment with anti-TNF therapy reversed these abnormalities in this T-cell subset though did not affect the frequency of IL-22^+^ T cells overall. Furthermore, blockade of IL-22 enhanced the IFNγ mediated release of CXCL-9. These results reveal CD4^+^ T-cell dysregulation in patients with PsA which can be reversed by anti-TNF and highlight the regulatory properties of IL-22 with important implications for therapeutic approaches that inhibit its production.

## Introduction

Psoriatic arthritis (PsA) is an inflammatory arthritis associated with psoriasis (1). TNF-α and IL-17 axis have been implicated in the pathogenesis of psoriasis and PsA (2, 3), although there is a stronger IL-17 signature in the skin compared with the synovium (4). Serum levels of a variety of cytokines including TNF, IL-17, and IL-22 have been positively correlated with psoriasis and PsA though the results have not always been consistent (5–9).

Interleukin-22 (IL-22) is member of the interleukin-10 (IL-10) family produced by lymphocytes but unlike other cytokines exerts its effects predominantly upon non-hemopoietin cells. IL-22 acts upon epithelial cells and fibroblasts at barrier sites such as the skin, lungs, gut, and synovium where it has been shown to be antiapoptotic, antimicrobial, pro-proliferative, and to promote cell survival and tissue regeneration (10). Dysregulation of IL-22 is thought to contribute to the development of a number of autoimmune diseases including psoriasis but also Crohn’s disease, and rheumatoid arthritis (11–13). IL-22 from dermal CD4^+^ T cells is highly expressed in lesional skin of psoriasis patients, and peripheral blood levels of IL-22 correlate strongly with disease activity, although these blood levels do not predict response to therapy (12). Relevant to psoriasis, IL-22 promotes keratinocyte proliferation, disrupts normal keratinocyte differentiation, and acts in concert with IL-17 to release pro-inflammatory cytokines and chemokines (2). Less is known about its role in inflamed joints of PsA.

While the role of IL-22 in psoriasis is generally considered to be uniformly pro-inflammatory, IL-22 also possesses regulatory and tissue protective properties in other settings (14). IL-22-deficient mice have increased susceptibility to hepatitis and similarly overexpression of the IL-22 gene in a mouse model of ulcerative colitis rescues the disease phenotype (15, 16). These protective effects are considered to be mediated through its action on target tissues rather than on dampening immune responses. To complicate matters further, IL-22 has both protective and pro-inflammatory effects in a number of diseases including asthma and collagen-induced arthritis depending upon the timing of administration (17–19). In a recent landmark paper, IL-22 was shown to have regulatory properties in inflammatory bowel disease (20).

In this study, we show that IL-22 is dysregulated in patients with PsA, particularly in the naïve CD4 T-cell compartment, and that IL-22 may operate as a regulatory cytokine to control inflammation.

## Materials and Methods

### Patients and Controls

We recruited patients with a diagnosis of PsA and healthy volunteers. Patients were either untreated, receiving a conventional disease modifying agent, or treated with anti-TNF therapy (adalimumab). See Table 1 for a summary of the clinical information. The study was approved by the UK NHS Health Research Authority, London City Road and Hampstead Committee, and written informed consent was obtained from all participants.

**Table 1:** Clinical features of the patients studied.

Samples	Healthy	PsA patients
Sample number total	41	83
**Sex**		
Male	8	39
Female	33	44
**Age**		
	35.4	49.3 ± 2.1
**Pattern of arthritis**		
Peripheral arthritis		65
Oligoarthritis		5
Axial involvement		9
Enthesitis		4
Active psoriasis (% patients)		67%
**Treatment type**		
Untreated		33
DMARD		30
Adalimumab		20
**Untreated patients**		
TJC		7.2 ± 1.76
SJC		4.5 ± 0.35
PASI		1.9 ± 0.63
ESR		15.4 ± 2.39
CRP		6.1 ± 1.45
DAS 28 ESR		3.3 ± 0.26
**DMARD patients**		
TJC		6.7 ± 2.64
SJC		2.1 ± 0.84
PASI		1.3 ± 0.35
ESR		16.5 ± 4.12
CRP		8.0 ± 1.42
DAS 28 ESR		2.8 ± 0.31
**Adalimumab patients**		
TJC		2.2 ± 0.62
SJC		1.9 ± 0.66
PASI		3.7 ± 1.97
ESR		8.4 ± 1.88
CRP		5.3 ± 2.78
DAS 28 ESR		2.3 ± 0.29

### Antibodies and Flow Cytometry

The following antibodies were used: Alexa Fluor 700-conjugated CD4 (clone RPA-TA; BD) or V500-conjugated CD4 (clone RPA-T4; BD); Alexa Fluor 700-conjugated-CD3 (clone OKT3; Biolegend); Alexa Fluor 488-conjugated CD3 (clone SP34-2; BD); Brilliant violet 711-conjugated CD8a (clone RPA-TA; BD); Alexa Fluor 488-conjugated IL-17A (clone BL168; Biolegend); PE Cy7 PE-Cyanine7-conjugated IL-22 (clone 22URTI eBioscience); V450-conjugated IFN-γ (clone B27, eBioscience); Brilliant Violet 605-conjugated CCR6 (Lone G034E3; Biolegend); PerCP/Cy5.5-conjugated CD194 (CCR4) (clone L291H4; Biolegend); PE-CF594-conjugated CD183 (CXCR3) (clone 1C6; BD); PE-conjugated CCR10 (clone 6588-5; eBioscience); PerCP-eFluor 710-conjugated Ki67 (clone 20Raj; eBioscience); APC-conjugated CD27 (clone O323; eBioscience); APC-H7-conjugated CD27 (clone M-T27; BD); PE-conjugated CD45RA (clone HI 100; BD); Alexa Fluor 700-conjugated CD45RA (clone Hi100, Biolegend); PE-Cy7-conjugated CD197 (CCR7) (clone 3D12; BD); FITC-conjugated CD62L (clone DREG-56; BD); PE-conjugated CXCL-9 (clone 49106; R&D systems); PE-conjugated mouse IgG1 isotype (clone 11711; R&D systems); Pacific blue™-conjugated mouse anti-STAT1 (pY701) (clone 14/P-STAT1); Pacific blue™-conjugated mouse IgG_1_κ isotype control (clone MOPC-21; BD); antihuman/mouse IL-22 functional grade purified (clone IL-22JOP; eBioscience); Rat IgG_2a_κ isotype control functional grade purified (clone eBR2a, eBioscience); antihuman IFNγ functional grade purified (NIB42 eBioscience); mouse IgG_1_κ isotype control functional grade purified (clone p3.6.2.8.1; eBioscience); soluble anti-CD3 (HIT-3a; eBioscience); and soluble anti-CD28 (CD28.2; eBioscience). All samples were acquired on an LSR II flow cytometer (BD) unless otherwise stated. Data were analyzed using FlowJo v10 software (TreeStar, Ashland, OR, USA).

### PBMC Isolation Surface Staining and Cell Culture

Whole peripheral blood mononuclear cells (PBMCs) were isolated by Ficoll-Paque (GE Healthcare) density gradient centrifugation using standard methods. Surface staining experiments were performed in accordance with manufacturer’s instructions. The eBioscience fixation/permeabilization buffer set was used for all intracellular staining experiments. The secretion of IL-22, IFNγ, and IL-17 was determined by flow cytometry after stimulation with soluble anti-CD3 (1 µg/mL) and anti-CD28 (1 µg/mL) for 3–5 days followed by 4-h stimulation with phorbol myristate acetate (PMA) (40 ng/mL) (Sigma), ionomycin (250 ng/mL) (Sigma), and Golgi-Stop™ 1 µg/mL (BD). LIVE/DEAD™ Fixable Blue Dead Cell Stain Kit was used to exclude dead cells.

### FACS and Culture of CD4^+^ T-Cell Subsets

Naïve (CD3^+^ CD4^+^ CD45RA^+^CD27^+^), central memory (CD3^+^ CD4^+^ CD45RA^−^ CD27^+^), and effector memory (CD3^+^ CD4^+^ CD45RA^−^ CD27^−^) T-cell subsets were isolated from whole PBMCs by flow cytometry (BD FACS Aria). Cell purity was evaluated after cell sorting by flow cytometry. Cells were then stimulated with soluble anti-CD3/anti-CD28 with or without recombinant IL-21 (30 ng/mL) (Peprotech) and cultured for 3–5 days. T-cell supernatants were taken for ELISA at culture day 3 in cells cultured without accessory cells. IL-22 and IFNγ secretion was measured at day 5 by flow cytometry as described.

### Ki67 Expression

Purified naïve (CD3^+^ CD4^+^ CD45RA^+^CD27^+^) T cells were stained for Ki67 expression at day 0 and following 5-day stimulation with soluble anti-CD3/anti-CD28 (1 µg/mL) and restimulation with PMA, ionomycin, and Golgi stop as outlined above.

### Cytokine Measurements by ELISA

ELISA kits measuring IL-22 (R&D systems) and IFNγ (eBioscience) were used to assay cytokines in culture supernatants in accordance with the manufacturer’s instructions.

### HaCaT Cell Culture and Chemokine Detection

HaCaT cells (a keratinocyte cell line) were cultured in Dulbecco’s Modified Eagle’s Medium (DMEM) (Sigma) supplemented with 5% fetal bovine serum and 0.5% penicillin/streptomycin. HaCaT cells were stimulated with either recombinant IFNγ (1 ng/mL) (Peprotech) and or recombinant IL-22 (30 ng/mL) (Peprotech) or day 5 culture supernatants from FACS sorted anti-CD3/anti-CD28 with or without IL-21 activated naïve (CD3^+^ CD4^+^ CD45RA^+^CD27^+^) T cells and cultured for 12 h with Golgi-Stop™. For blocking experiments, antihuman IFNγ antibody (10 µg/mL) or antihuman/mouse IL-22 antibody (10 ng/mL) or their respective isotype controls (10 ng/mL) were used. Adherent HaCaT cells harvested with 0.25% trypsin- dthylenediaminetetraacetic acid (Gibco) and intracellular CXCL-9 production measured by flow cytometry as described above.

### Phosphoflow STAT1

HaCaT cells were stimulated with recombinant IL-22 (50 ng/mL) and/or recombinant IFNγ (0.5 ng/mL) for 15 min. Cells were washed and fixed with 2% paraformaldehyde, then washed with cold 1× phosphate-buffered saline before resuspending in ice cold methanol at 4°C for 30 min. Cells were washed in magnetic-activated cell sorting buffer and stained intracellularly with anti-STAT1 antibody or anti-STAT1 isotype for 60 min at room temperature. The expression of pSTAT1 in HaCaT cells was then measured immediately by flow cytometry.

### Statistical Analysis

Statistical analysis was performed using GraphPad Prism version 5 (GraphPad Software, San Diego, CA, USA). Data were assessed for normality using the Kolmogorov–Smirnof test. Where data were normally distributed paired or unpaired student *t*-test was used unless three independent comparisons applied when one-way analysis of variance (ANOVA) with Tukey’s *post hoc* analysis was used. Where data were not normally distributed, the non-parametric Kruskal–Wallis (with Dunn’s post-test analysis) was used. A *p*-value of <0.05 was considered significant.

## Results

### The Frequency of CD4^+^ IL-22^+^ Cells Reduced in the Peripheral Blood of PsA Patients Compared with Healthy Individuals

**Figure 1:**
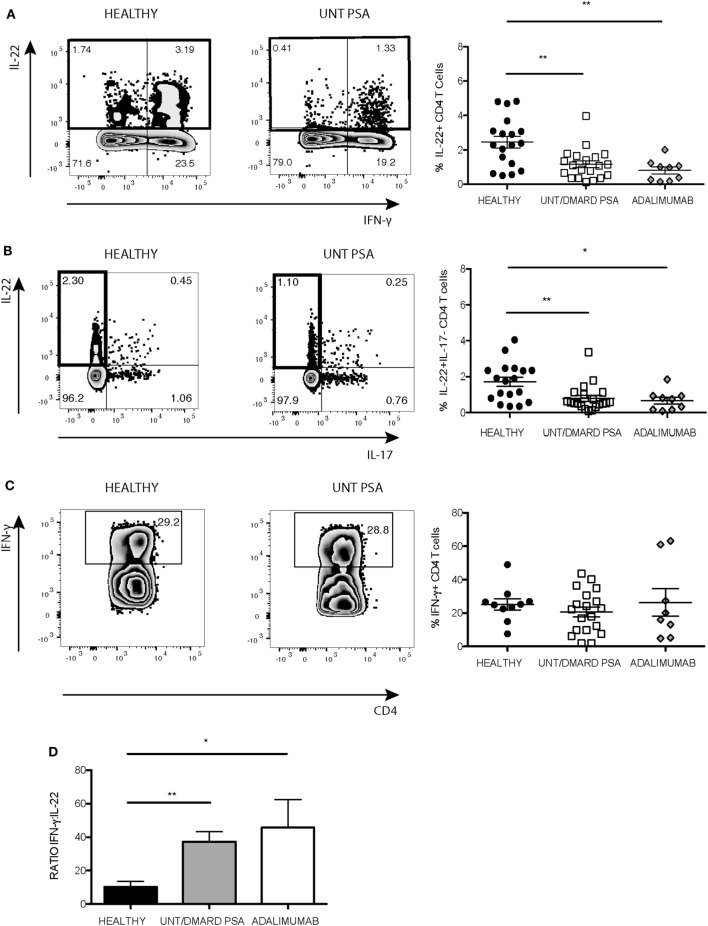
Frequency of IL-22^+^ CD4^+^ T cells reduced in PsA compared with healthy controls. PBMCs were stimulated with anti-CD3/anti-CD28 and the production of IL-22 and IFNγ quantified using flow cytometry. Representative flow cytometry and cumulative data showing frequency of **(A)** CD4^+^IL-22^+^ T cells and **(B)** IL-22^+^IL-17^−^ CD4^+^ T cells in healthy controls (healthy) (*n* = 18), untreated/DMARD PsA patients (untreated/DMARD PsA) (*n* = 20), and adalimumab treated patients (*n* = 9). **(C)** Representative flow cytometry and cumulative data showing frequency of IFNγ^+^CD4^+^ T cells in healthy (*n* = 10), Untreated/DMARD PsA (*n* = 20) and adalimumab treated patients (*n* = 8). *p < 0.05; **p < 0.01 one-way ANOVA with Tukey’s *post hoc* analysis. Error bars represent mean ± SE. **(D)** Bar graph showing cumulative total IFNγ/IL-22 ratio in CD4^+^ T-cell Kruskal–Wallis test with Dunn’s *post hoc* analysis *p < 0.05; **p < 0.01. Error bars represent mean ± SE. ANOVA, analysis of variance; DMARD, disease-modifying antirheumatic drug; IFNγ, interferon-gamma; IL-22, interleukin-22; PBMCs, peripheral blood mononuclear cells; PsA, psoriatic arthritis.

The production of IL-22 and IFNγ was quantitated in activated CD4^+^ T cells from patients with PsA. The frequency of CD4^+^IL-22^+^ T cells was less in PsA compared with healthy controls irrespective of therapy including with the TNF inhibitor adalimumab (Figure 1A). The percentage of Th22 cells (IL-22^+^IL-17^−^ CD4^+^ T cells) was also reduced (Figure 1B). In contrast, the percentage of CD4^+^IFNγ^+^ T cells was similar between PsA patients and healthy controls (Figure 1C) resulting in an increased ratio of IFNγ:IL-22 in CD4^+^ T cells in PsA compared with healthy controls (Figure 1D).

**Figure 2:**
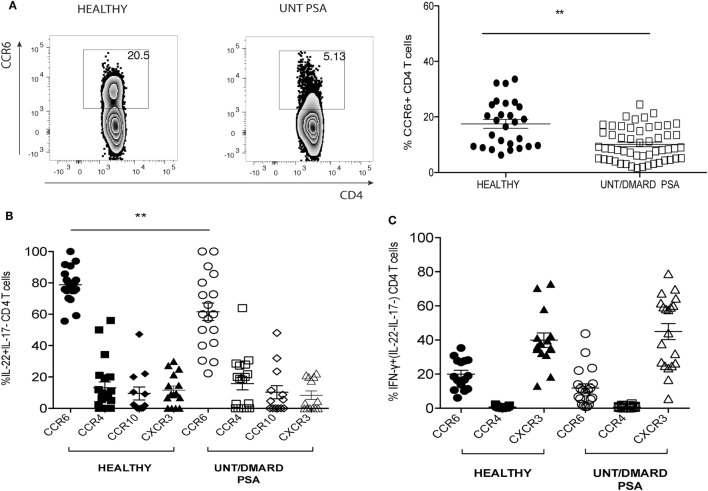
Frequency of CD4^+^CCR6^+^IL-22^+^ T cells in PsA reduced as compared with healthy controls. **(A)** Representative FACS plot and cumulative scatter plot showing percentage CCR6^+^CD4^+^ T cells in healthy (*n* = 27) and untreated/DMARD PsA (*n* = 53). **(B)** Cumulative dot plot showing CCR6, CXCR3, CCR4, and CCR10 expression on IL-22^+^IL17^−^IFNγ^−^ CD4^+^ T cells; healthy (*n* = 20) and untreated/DMARD PsA (*n* = 18). **(C)** Cumulative dot plot showing CCR6, CXCR3, and CCR4 expression on IFNγ^+^IL-22^−^IL-17^−^ CD4^+^ T cells; healthy (*n* = 15) and untreated/DMARD PsA (*n* = 20). **p < 0.01 unpaired Student’s *t*-test. Error bars represent mean ± SE. DMARD, disease-modifying antirheumatic drug; FACS, fluorescence-activated cell sorting; IFNγ, interferon-gamma; IL-22, interleukin-22; PsA, psoriatic arthritis.

T helper-22 cells express the chemokine receptors CCR6, CCR4, and CCR10. CCR6 expression was globally reduced on CD4^+^ T cells of PsA patients compared with healthy controls (Figure 2A). The majority of IL-22^+^ IL-17^−^ CD4^+^ T cells in the healthy control samples expressed CCR6, but this was significantly less in PsA patients (Figure 2B). CCR4, CCR10, and CXCR3 were expressed at much lower frequencies on IL-22^+^ CD4^+^ T cells (~10–20%) without any differences between patients and controls (Figure 2B). In contrast to the reduction in CCR6 expression on IL-22^+^ CD4^+^ T cells, there was no change in CXCR3 expression on T helper-1 (Th1) cells (Figure 2C).

### IFNγ and IL-22 Production Increased in Activated “Naïve” PsA T Cells

**Figure 3:**
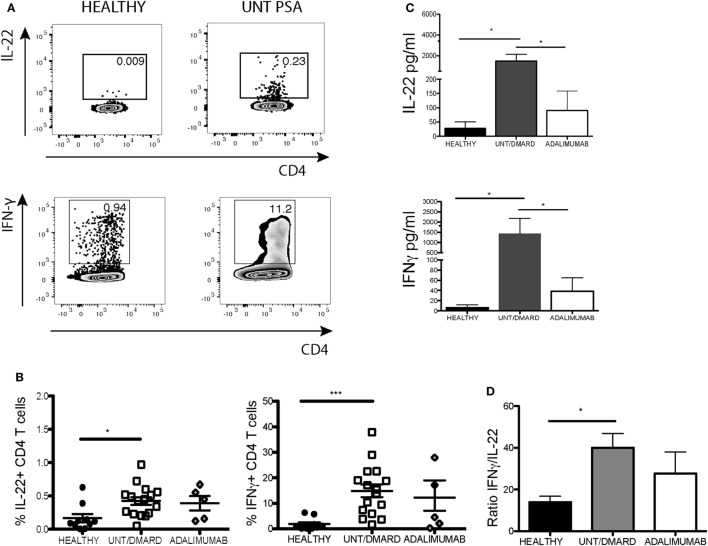
Activated naïve CD4^+^ T cells from PsA patients producing more IL-22 and IFNγ compared with healthy controls. Purified naïve (CD3^+^CD4^+^CD45RA^+^CD27^+^) T cells were stimulated with anti-CD3/anti-CD28. After 5-day culture, cells were restimulated with PMA/ionomycin and intracellular IL-22 and IFNγ production evaluated. Supernatants were taken at day 3 for quantitative ELISA. **(A)** Representative flow cytometry plot and **(B)** cumulative data showing frequency of IL-22^+^ and IFNγ^+^ CD4^+^ T cells, healthy (*n* = 10), untreated/DMARD PsA (*n* = 16), and adalimumab-treated PsA (*n* = 5) patients. **(C)** Concentration of IL-22 and IFNγ in day 3 naïve T-cell supernatants: healthy (*n* = 8), untreated/DMARD PsA patients (*n* = 10), and adalimumab-treated PsA patients (*n* = 5). **(D)** Bar graph showing cumulative IFNγ/IL-22 ratio in the purified naïve T cells based on flow cytometry staining: healthy (*n* = 10), untreated/DMARD PsA (*n* = 16), and adalimumab-treated PsA patients (*n* = 5) *p < 0.05; **p < 0.01; ***p < 0.001; Kruskal–Wallis test with Dunn’s *post hoc* analysis (error bars represent mean ± SE). DMARD, disease-modifying antirheumatic drug; IFNγ, interferon-gamma; IL-22, interleukin-22; PMA, phorbol myristate acetate; PsA, psoriatic arthritis.

Next we investigated which T-cell subset contributed to IL-22 dysregulation. T-cell subsets were classified as naïve (CD3^+^CD4^+^CD27^+^CD45RA^+^), central memory (CD3^+^CD4^+^CD27^+^CD45RA^−^), and effector memory (CD3^+^CD4^+^CD27-CD45RA^−^) cells based on the presence or absence of the surface molecules CD45RA and CD27 (21). The percentage of IL-22^+^ CD4^+^ T cells was reduced in activated central memory CD4^+^ T cells of PsA patients without any significant difference in IFNγ expression or in the ratio of IL-22:IFNγ (Figure S1 in Supplementary Material). The production of both these cytokines was undisturbed from activated effector CD4 memory cells (Figures S1A,B in Supplementary Material). Unexpectedly, the frequency of IL-22^+^ and IFNγ^+^ naïve CD4^+^ T cells were significantly elevated in PsA patients compared with healthy controls (Figures 3A,B). Quantification of IL-22 and IFNγ levels in day 3 culture supernatants revealed markedly increased secretion of both these cytokines from naïve T cells, which was lessened in patients treated with adalimumab (Figure 3C). The IFNγ:IL-22 ratio from these unconventional naïve T cells more than doubled in PsA patients compared with healthy controls with amelioration of these changes following anti-TNF therapy (Figure 3D). There was no significant correlation between the skin or joint disease activity scores and the percentage of naïve T cells or the ratio of IFNγ:IL-22 production from these unconventional naïve T cells in PsA (data not shown).

### Activated Naïve CD4^+^ T Cells Showing Features of Memory Cells and Promoting CXCL-9 Production from HaCaT Keratinocytes

**Figure 4:**
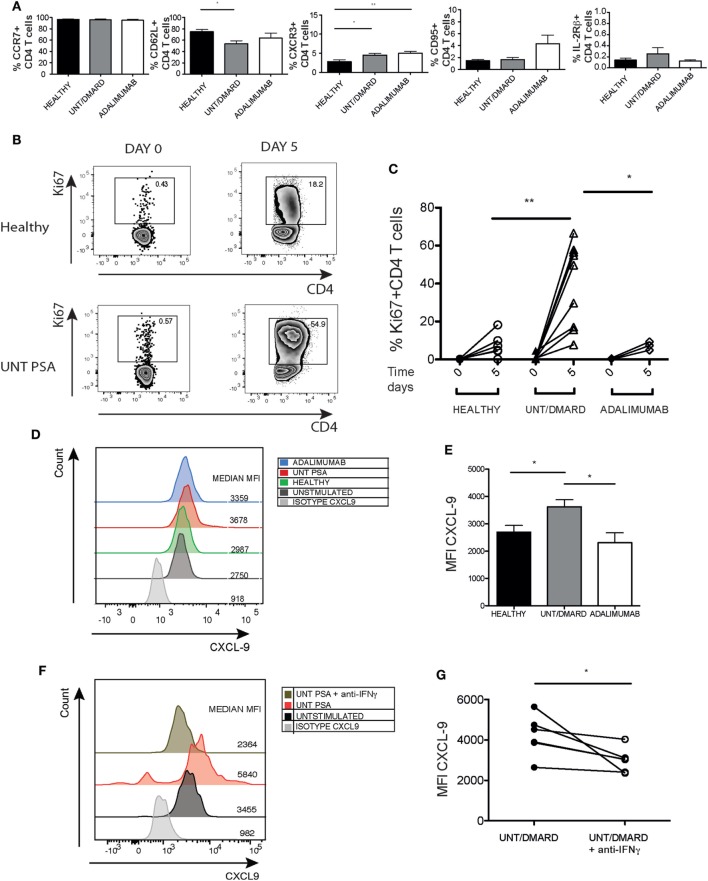
The unconventional naïve CD4^+^ T cells from PsA patients exhibiting some phenotypic and functional features of memory cells and promoting CXCL9 expression from HaCaT keratinocytes. PBMCs were surface stained for CCR7, CD62L, CXCR3, CD95, and IL-2Rβ and percentage expression on naïve (CD3^+^CD4^+^CD45RA^+^CD27^+^) T cells evaluated. **(A)** Frequency of CCR7^+^, CD62L^+^, CXCR3^+^, CD95^+^, and IL-2Rβ^+^ cells in healthy (*n* = 11–16): untreated/DMARD PsA (*n* = 19–32) and adalimumab-treated PsA (*n* = 6–8) patients. *p < 0.05 Kruskal–Wallis test with Dunn’s *post hoc* analysis. Error bars represent mean ± SE. **(B,C)** Naïve (CD3^+^CD4^+^CD45RA^+^CD27^+^) T cells were purified and Ki67 expression measured at baseline and after 5-day stimulation with anti-CD3/anti-CD28. Representative flow cytometry plot and cumulative graph showing frequency of CD4^+^Ki67^+^ T cells at baseline and after stimulation in healthy (*n* = 6), untreated/DMARD PsA (*n* = 9) and adalimumab-treated PsA (*n* = 4) patients. *p < 0.05; **p < 0.01 unpaired Student’s *t*-test. HaCaT cells were stimulated with day 5 activated T-cell supernatants with or without anti-IFNγ from healthy, untreated/DMARD PsA, and adalimumab-treated PsA patients. **(D)** Representative histogram depicting MFI of CXCL-9 expression in HaCaT cells. **(E)** Bar graph showing CXCL-9 MFI expression in stimulated HaCaT cells, healthy (*n* = 6), untreated/DMARD PsA patients (*n* = 11), and adalimumab-treated PsA patients (*n* = 4) *p < 0.05 unpaired Student’s *t*-test. Error bars represent mean ± SE. **(F,G)** Representative histogram and cumulative data showing MFI CXCL-9 expression in HaCaT cells stimulated with supernatant with or without anti-IFNγ from untreated/DMARD PsA activated naïve CD4^+^ T cells (*n* = 5) *p < 0.05 paired Student’s *t*-test. DMARD, disease-modifying antirheumatic drug; IFNγ, interferon-gamma; IL-22, interleukin-22; MFI, median fluorescent intensity; PBMCs, peripheral blood mononuclear cells; PsA, psoriatic arthritis.

Studies have shown that a population of long-lived memory T cells with characteristics of naïve T cells exists with self-renewal properties and the ability to repopulate the central memory, effector memory, and effector T-cell pools (22, 23). Prompted by the aberrant cytokine producing properties of ostensibly naïve CD4 T cells from PsA patients, we further characterized this population with respect to CCR7, CD62L, CXCR3, CCR6, CD95 (Fas), and IL-2 receptor beta (IL-2Rβ) expression. Greater than 95% of CD27^+^CD45RA^+^CD4^+^ T cells in healthy controls and PsA patients expressed the naïve T-cell marker CCR7 (Figure 4A). However, the percentage of CD27^+^CD45RA^+^CD4^+^ T cells expressing the lymph node homing lectin CD62L was reduced in PsA patients compared with healthy controls with a similar trend in anti-TNF-treated patients (Figure 4A). Furthermore, there was a significant increase in CXCR3 expression in naïve T cells from PsA patients compared with healthy controls (Figure 4A). The expression of both CD95 and IL-2Rβ were low in the CD27^+^CD45RA^+^CD4^+^ T-cell population.

We next evaluated the proliferative capacity of these unconventional CD27^+^CD45RA^+^CD4^+^ naïve T cells. *Ex vivo* Ki67 expression was similar between healthy controls, PsA patients, and adalimumab-treated PsA patients (Figures 4B,C). However, upon stimulation the unconventional naïve T cells from PsA patients had a far greater proliferative capacity compared with naïve T cells from healthy controls which was fully reversed in anti-TNF-treated patients (Figures 4B,C).

An *in vitro* model of inflammation was utilized to assess the impact of IL-22 and IFNγ dysregulation in the CD27^+^CD45RA CD4^+^ unconventional naïve T-cell subset. Culture supernatants from the unconventional naïve T cells isolated from PsA patients promoted higher expression of the Th1 chemokine CXCL-9 by HaCaT cells (a keratinocyte cell line) after short-term culture compared with healthy controls and patients treated with anti-TNF therapy (Figures 4D,E). CXCL-9 production stimulated by the unconventional naïve T-cell supernatants was inhibited by an IFNγ-blocking antibody (Figures 4F,G).

### IL-22 Regulating IFNγ-Mediated CXCL9 Release from HaCaT Cells Stimulated by Naïve CD4^+^ T Cells from PsA Patients

**Figure 5:**
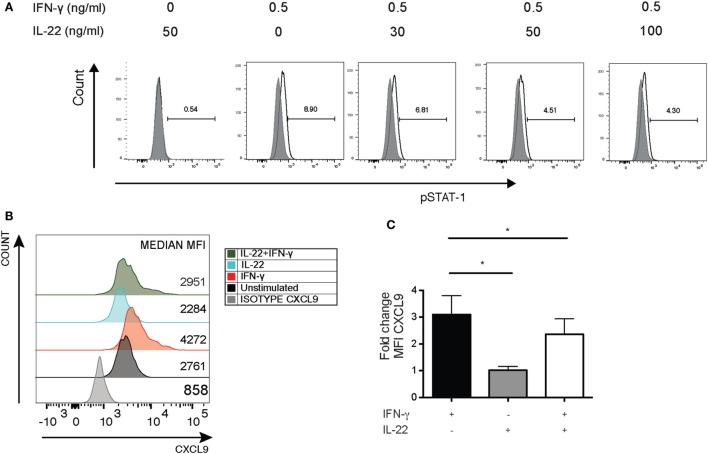
IL-22 suppressing IFNγ-driven pSTAT1 and CXCL-9 production in HaCaT keratinocytes. HaCaT keratinocytes were cultured for 15 min with different concentrations of recombinant IL-22 but with a fixed concentration of IFNγ (0.5 ng/mL). pSTAT1 expression was detected by flow cytometry. Alternatively, HaCaT cells were stained for intracellular CXCL-9 expression. **(A)** Representative histogram showing pSTAT1 expression in HaCaT cells (representative of four independent experiments). **(B,C)** Representative histogram showing MFI for CXCL9 expression and bar graph depicting cumulative fold change in CXCL9 expression in HaCaT cells after stimulation with IL-22 (30 ng/mL) and/or IFNγ (1 ng/mL) (*n* = 5). *p < 0.05 unpaired Student’s *t*-test. Error bars represent mean ± SE. IFNγ, interferon-gamma; IL-22, interleukin-22; MFI, median fluorescent intensity.

To investigate whether there was a relationship between IFNγ and IL-22, we initially cultured HaCaT cells with recombinant IL-22 (rIL-22) and/or IFNγ (rIFNγ). IL-22 suppressed IFNγ-driven STAT1 phosphorylation (Figure 5A) and the ability of rIFNγ to induce CXCL-9 (Figures 5B,C).

**Figure 6:**
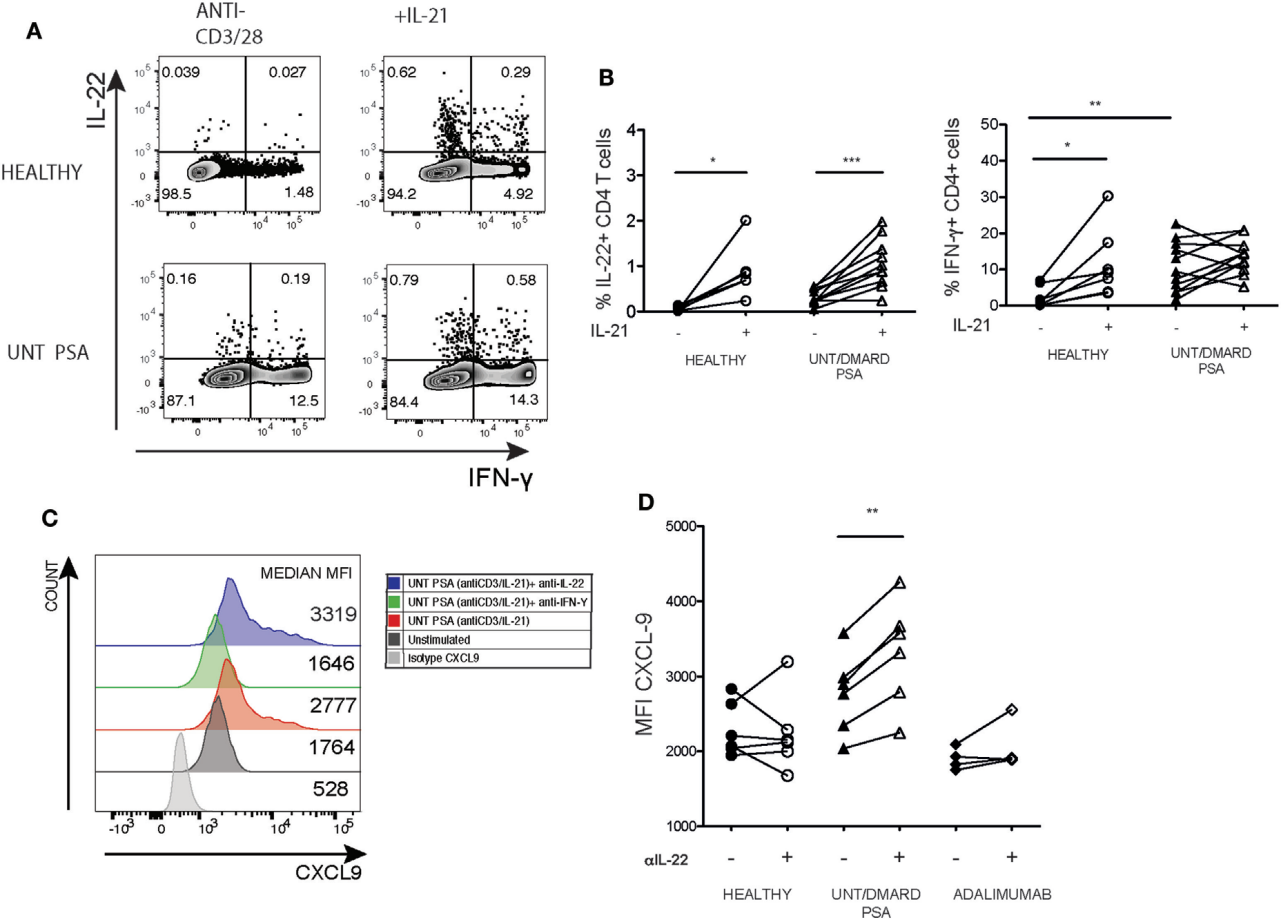
Blockade of IL-22 in supernatants from the unconventional naïve T cells from PsA patients further elevates IFNγ driven CXCL-9 production in HaCaT keratinocytes. Purified naïve T cells were activated with anti-CD3/anti-CD28 with or without recombinant IL-21 (30 ng/mL) for 5 days before assaying intracellular cytokine production. **(A)** Representative flow cytometry depicting the frequency of IL-22^+^ and IFNγ^+^ CD4 T cells in PsA and healthy. **(B)** Cumulative paired expression of IL-22 and IFNγ in anti-CD3/anti-CD28 and anti-CD3/anti-CD28^+^ IL-21 stimulated cells; healthy (*n* = 6), untreated/DMARD PsA (*n* = 10) IFNγ: healthy (*n* = 8), untreated/DMARD PsA (*n* = 11). *p < 0.05; **p < 0.01; ***p < 0.001 paired Student’s *t*-test. HaCaT cells were stimulated with day 5 supernatants from anti-CD3/CD28/IL-21 activated naïve CD4^+^ T cells with or without anti-IFNγ or anti-IL-22 for 12 h in the presence of Golgi Stop and intracellular CXCL-9 production evaluated. **(C)** Representative histogram showing mean fluorescent intensity of CXCL-9 expression in HaCaT cell stimulated with supernatant from Unt PsA naïve CD4^+^ T cells with or without anti-IFNγ or anti-IL-22. **(D)** Plot showing cumulative MFI CXCL9 in presence or absence of anti-IL-22; healthy (*n* = 6) untreated/DMARD PsA (*n* = 6), adalimumab (*n* = 4); **p < 0.01 paired Student’s *t*-test. DMARD, disease-modifying antirheumatic drug; IFNγ, interferon-gamma; IL-22, interleukin-22; MFI, median fluorescent intensity; PsA, psoriatic arthritis.

The cytokine IL-21 can trigger IL-22 production in CD4^+^ T cells (24). We thus investigated whether IL-21 promoted IL-22 production in activated unconventional naïve PsA CD4^+^ T cells and what functional effects increasing IL-22 availability would have on CXCL9 production. After 5-day culture, IL-21 increased IL-22 production by naïve CD4^+^ T cells from both healthy controls and PsA patients (Figures 6A,B). However, IL-21-driven expansion of IFNγ^+^ T cells only occurred in healthy individuals (Figures 6A,B). We then assessed the functional consequences of increased IL-22 production. Inhibition of IL-22 increased CXCL-9 expression driven by supernatants from the unconventional naïve CD4 T cells isolated from patients with PsA but not from healthy controls or patients treated with adalimumab (Figures 6C,D).

## Discussion

We found that the frequency of IL-22 producing CD4 T cells following activation *in vitro* is reduced in PsA patients compared with healthy controls, whereas the percentage of CD4^+^IFNγ^+^ remained stable. This reduction of IL-22 expressing CD4^+^ T cells is principally accounted for by changes in the central memory CD4^+^ T-cell compartment. Comparative data on IL-22 expression in peripheral CD4^+^ T cells from PsA and healthy controls are limited with conflicting results from peripheral blood and synovial fluid (6, 25, 26). The reduced frequency of CCR6^+^ IL-22^+^ CD4^+^ cells in the peripheral blood of PsA patients could be explained by their migration to sites of inflammation possibly through a CCR6-dependent mechanism. About two-thirds of our patients also had psoriasis, though mostly minimal disease (Table 1), and therefore we cannot distinguish the immune consequences of inflammatory joint from inflammatory skin disease, nor determine to which inflammatory site the IL-22^+^ cells would be directed toward.

The most surprising finding with respect to IL-22 production by CD4^+^ T cells in patients with PsA occurred within the “naïve” T-cell compartment. Significant polarization in this unconventional naïve subset was associated with a twofold increase in the ratio of IFNγ^+^:IL-22^+^ production, which was greater than in the other CD4^+^ T-cell subsets underscoring the pro-inflammatory potential of naïve CD4 T cells in PsA. Alterations in the phenotype of these unconventional naïve CD4 T cells in PsA suggest adoption of memory properties similar to that observed following infection (23) but are also present in healthy individuals at low frequencies (27). Moreover, these T cells with a naïve phenotype produce effector cytokines and respond rapidly to stimulation and primed to differentiate into effector and memory cells, thereby contributing to disease exacerbations. Like conventional naïve CD4^+^ T cells, these memory cells with a naïve phenotype can circulate between the blood and lymphoid tissue but their lower expression of CD62L together with increased expression of CXCR3 would enhance migration to sites of inflammation (28). Although the unconventional naïve T cells described here did not express CD95 or IL-2Rβ which would suggest that they are distinct from antigen-experienced cells with memory stem cell-like properties, the high proliferative response and somewhat increased CXCR3 expression are characteristic of this T-cell population (22, 29). Alternatively, these PsA unconventional naïve T cells could be closer to the more recently described naïve T cells with a memory cell phenotype which do not express CD95 (23). The majority of the abnormalities observed with this unconventional naïve CD4 subset such as the enhanced proliferative capacity and ability to increase CXCL-9 production were reversed by anti-TNF therapy, suggesting that a TNF-dependent mechanism contributes to these changes in PsA. In contrast, anti-TNF did not reverse the lower percentage of total and central memory IL-22 expressing CD4 T cells in patients with PsA. Thus, it appears that anti-TNF principally targets the aberrant cytokine production and proliferative response of the dysregulated naïve CD4 T cells in PsA rather than other CD4 T-cell subsets.

It has previously been shown that the Th-17 cytokine IL-21 stimulates IL-22 release in CD4^+^ T cells (24). We confirmed that IL-21 promoted IL-22 production in activated naive CD4^+^ T cells of PsA patients and healthy controls. In healthy individuals, but not patients with PsA, IL-21 also induced increased IFNγ expression. There is controversy as to whether IL-21 modulates CD4 T-cell IFNγ production; we speculate that increased IL-21, which occurs in PsA, dampens the IFNγ but not the IL-22 response to IL-21, which was added in activated naïve T-cell cultures (24, 30). Naïve T-cell-derived culture supernatants, activated in the presence of IL-21 to enhance IL-22 production, induced CXCL-9 production from HaCaT cells. IL-22 blockade further enhanced CXCL9 production from HaCaT cells. These data highlight a potential regulatory role for IL-22 in PsA, in particular inhibition of the production of CXCL9 which has been implicated in the pathogenesis of PsA (31). Our findings are consistent with the suppression of CXCL-9 by IL-22 in an animal model of lung fibrosis which limited recruitment of CD4^+^CXCR3^+^ T cells and attenuation of lung inflammation and fibrosis (32). More recently, anti-TNF therapy has been shown to be effective in inflammatory bowel disease in part through suppressing IL-22-binding protein thereby implicating IL-22 as a protective cytokine (20). Thus, IL-22 is emerging as a key regulatory cytokine in a number of different diseases. However, contrasting these reports IL-22 has frequently been identified as pro-inflammatory in a range of diseases including psoriasis, though little is understood about its role in PsA. For instance, low levels of IL-22 binding protein and high IL-22 concentrations are associated with worse inflammation in psoriasis (33). Moreover, IL-22 induces psoriasis like skin changes and its blockade can be as effective as anti-TNF in a psoriasis model (34). The fact that efforts to target IL-22 in patients with psoriasis have largely stalled perhaps attests to the challenges in targeting this cytokine which has potentially opposing roles often in the same disease.

## Ethics Statement

The study was approved by the UK NHS Health Research Authority, London City Road and Hampstead Committee, and written informed consent was obtained from all participants in accordance with the Declaration of Helsinki.

## Author Contributions

AE, HB, MV-C, and ME designed the experiments. AE and HB conducted the experiments. AE, HB, MV-C, and ME undertook the analysis. AE and ME wrote the paper with input from all other authors.

## Conflict of Interest Statement

The authors declare that the research was conducted in the absence of any commercial or financial relationships that could be construed as a potential conflict of interest.

## Supplementary Material

Figure S1 **(A)** IL-22 and IFNγ production from purified central memory (CD3^+^CD4^+^CD45RA^−^CD27^+^) T cells healthy (*n* = 13), untreated/DMARD PsA *n* = 17, adalimumab-treated PsA (*n* = 5) and effector memory (CD3^+^ CD4^+^CD45RA^−^CD27^−^) T cells, healthy (*n* = 9), untreated/DMARD PsA (*n* = 12), adalimumab-treated PsA (*n* = 4) after 5-day stimulation with anti-CD3/anti-CD28. **(B)** IFNγ:IL-22 ratio in central memory cells and effector memory T cells.
