# Supplementary figures and images for: CD4 T-Cell Dysregulation in Psoriatic Arthritis Reveals a Regulatory Role for IL-22
Source: Front Immunol. 2017 Oct 27;8:1403. doi: 10.3389/fimmu.2017.01403 (PMC5666299; doi:10.3389/fimmu.2017.01403)

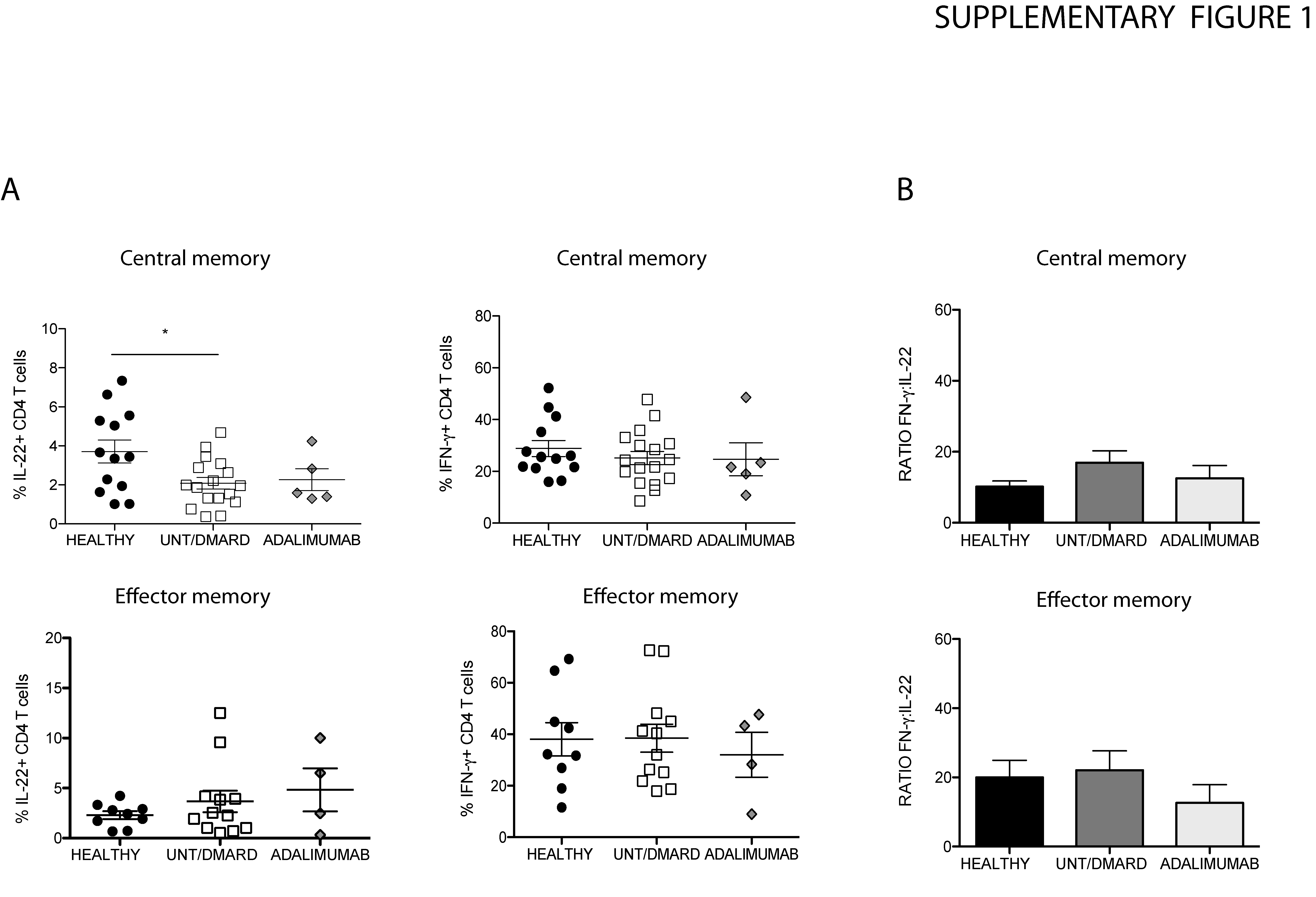

Supplement: Figure S1 — (A) IL-22 and IFNγ production from purified central memory (CD3+CD4+CD45RA−CD27+) T cells healthy (n = 13), untreated/DMARD PsA n = 17, adalimumab-treated PsA (n = 5) and effector memory (CD3+ CD4+CD45RA−CD27−) T cells, healthy (n = 9), untreated/DMARD PsA (n = 12), adalimumab-treated PsA (n = 4) after 5-day stimulation with anti-CD3/anti-CD28. (B) IFNγ:IL-22 ratio in central memory cells and effector memory T cells. [file image_1.tif]
